# A retrospective cohort study on the risk of stroke in relation to *a priori* health knowledge level among people with type 2 diabetes mellitus in Taiwan

**DOI:** 10.1186/s12872-017-0568-4

**Published:** 2017-05-22

**Authors:** Yun-Ju Lai, Hsiao-Yun Hu, Ya-Ling Lee, Po-Wen Ku, Yung-Feng Yen, Dachen Chu

**Affiliations:** 1 School of Medicine, National Yang-Ming University, Taipei, Taiwan; 2 Division of Endocrinology and Metabolism, Department of Internal Medicine, Puli Branch of Taichung Veterans General Hospital, Nantou, Taiwan; 3 Department of Exercise Health Science, National Taiwan University of Sport, Taichung, Taiwan; 4 Department of Education and Research, Taipei City Hospital, Taipei, Taiwan; 5 Institute of Public Health and Community Medicine Research Center, National Yang-Ming University, Taipei, Taiwan; 6 Department of Dentistry, Taipei City Hospital, Taipei, Taiwan; 7 Department of Dentistry, School of Dentistry, National Yang-Ming University, Taipei, Taiwan; 8 Graduate Institute of Sports and Health, National Changhua University of Education, Changhua, Taiwan; 9 Section of Infectious Diseases, Taipei City Hospital, Taipei City Government, No.145, Zhengzhou Rd., Datong Dist., Taipei, 10341 Taiwan; 10 Department of Health and Welfare, College of City Management, University of Taipei, Taipei, Taiwan; 11 Center for Infectious Disease and Cancer Research, Kaohsiung Medical University, Kaohsiung, Taiwan; 12 Department of Health Care Management, National Taipei University of Nursing and Health Sciences, Taipei, Taiwan; 13 Department of Neurosurgery, Taipei City Hospital, Taiwan, No.145, Zhengzhou Rd., Datong Dist., Taipei, 10341 Taiwan; 14 Faculty of Medicine, School of Medicine, National Yang-Ming University, Taipei, Taiwan

**Keywords:** Type 2 diabetes mellitus, Stroke, Health knowledge, Retrospective cohort study, Taiwan

## Abstract

**Background:**

Intervention of diabetes care education with regular laboratory check-up in outpatient visits showed long-term benefits to reduce the risk of macrovascular complications among people with type 2 diabetes. However, research on the level of a priori health knowledge to the prevention of diabetic complications in community settings has been scarce. We therefore aimed to investigate the association of health knowledge and stroke incidence in patients with type 2 diabetes in Taiwan.

**Methods:**

A nationally representative sample of general Taiwanese population was selected using a multistage systematic sampling process from Taiwan National Health Interview Survey (NHIS) in 2005. Subjects were interviewed by a standardized face-to-face questionnaire in the survey, obtaining information of demographics, socioeconomic status, family medical history, obesity, health behaviors, and 15-item health knowledge assessment. The NHIS dataset was linked to Taiwan National Health Insurance claims data to retrieve the diagnosis of type 2 diabetes in NHIS participants at baseline and identify follow-up incidence of stroke from 2005 to 2013. Univariate and multivariate Cox regressions were used to estimate the effect of baseline health knowledge level to the risk of stroke incidence among this group of people with type 2 diabetes.

**Results:**

A total of 597 diabetic patients with a mean age of 51.28 years old and nearly half of males were analyzed. During the 9-year follow-up period, 65 new stroke cases were identified among them. Kaplan–Meier curves comparing the three groups of low/moderate/high knowledge levels revealed a statistical significance (*p*-value of log-rank test <0.01). After controlling for potential confounders, comparing to the group of low health knowledge level, the relative risk of stroke was significantly lower for those with moderate (adjusted hazard ratio [AHR] = 0.63; 95% CI, 0.33–1.19; *p*-value = 0.15) and high level of health knowledge (AHR = 0.43; 95% CI, 0.22–0.86; *p*-value = 0.02), with a significant linear trend (*p*-value = 0.02).

**Conclusions:**

An exposure-response gradient of moderate to high health knowledge levels to the prevention of stroke incidence among people with type 2 diabetes in community was found with 9 years of follow-up in Taiwan. Development and delivery of health education on stroke prevention to people with type 2 diabetes are warranted.

## Background

Type 2 diabetes is a common chronic disease and, without proper controls, almost inevitably leads to costly microvascular and macrovascular complications. In Taiwan, the direct costs of healthcare for diabetes represented 11.5% of all health expenditure in Taiwan and were 4.3 times the average costs taken by non-diabetic health expenditure [1]. Diabetes accounts for nearly 14% of total healthcare expenditures, at least half of which are related to microvascular or macrovascular complications, including retinopathy, foot ulcer, myocardial infarction, stroke, and end-stage renal disease [2].

Reduction of other risk factors was found to decrease the risk of macrovascular complications for people with diabetes mellitus. Approaches to reducing such risk factors include smoking cessation, aggressive management of hypertension and hyperlipidemia, and prophylaxis use of aspirin in people with or at a high risk of cardiovascular diseases [3–6]. People with diabetes are encouraged to participate in a comprehensive education program for diabetes mellitus self-management, which includes nutrition guidance, elevation of physical activity, optimal glycemic control and prevention of complications [7, 8]. Previous studies comparing diabetes education with routine care had shown a small but statistically significant glycated hemoglobin reduction in patients receiving diabetes education [9]. In Taiwan, a study reported that “pay-for-performance” (P4P) programs for diabetes care in clinical settings showed potential long-term benefits on reducing risks of macrovascular complications [10]. In Hong Kong, the Enrolment to Patient Empowerment Programme significantly reduced all-cause mortality and delayed first macrovascular or microvascular disease events in obese type 2 diabetic patients [11]. However, subjects involved in such intervention programs could be highly selective, resulting in restricted generalizability. Prior studies reported that implementation of community-based education with a customized curriculum can improve the health knowledge and behaviors among healthy residents in community and accordingly reduce cardiovascular disease risks [12, 13]. However, no evidence has been shown about if the improved health knowledge really worked on the prevention of cardiovascular diseases. The association of a priori awareness of health knowledge as a major research interest and risks of incident stroke among a high-risk group like people with diabetes in community settings has never been studied. We thus aimed to examine the association of health knowledge at baseline in a national survey and the following risk of incident stroke in people with diabetes in Taiwan.

## Methods

### Study setting and data source

We investigated the association between health knowledge and incidence of first stroke in people with diabetes aged 18 to 65 years with a retrospective cohort design. In 2005, the National Health Interview Survey (NHIS) in Taiwan managed to retrieve a representative sample from the general population of Taiwan. The multistage-stratified sampling scheme was based on the extent of urbanization, geographic areas, and boundaries of local administration. NHIS data were collected by face-to-face interviews with a standard protocol. The survey covered information on demographics, socioeconomic status, height and weight, health knowledge and related behaviors. Survey respondents with a consent provided were then linked to Taiwan National Health Insurance (NHI) system claim data from 2005 to 2013. The NHI database includes all outpatient visits, hospital admissions, costs, date of procedures, International Classification of Diseases, Ninth Revision, Clinical Modification (ICD-9-CM) codes, and drug records. The follow-up period started on the date of interview for health knowledge and ended at stroke incidence, date of death, or end of follow-up period (Dec 31, 2013), whichever came earlier.

### Covariates

#### Major exposure of interest

The questionnaire of health knowledge consisted of 15 items on knowledge of preventing diabetes mellitus, hypertension, and chronic kidney disease. Knowledge scores ranged from 0 to 15. Participants were asked, “How can a person reduce the risks of developing chronic diseases?” for yes/no options of 1) Avoiding too intensive physical exercise, 2) Not taking medicines not prescribed by doctors, 3) Minimizing the intake of salty food, 4) Controlling blood pressure and glucose levels if diagnosed, 5)Taking regular health examinations, 6) Drinking sufficient amount of water on daily basis, 7) Doing urination as often as needed, 8) Maintaining ideal body weight, 9) No smoking, 10) Being abstinent from alcohol, 11) Doing exercise regularly, 12) Consuming healthy food, 13) Minimizing stress, 14) Consulting medical professionals whenever needed, and 15) Others (open answers). Each correct response accounts for one point. Of the 597 participants, 77 (12.90%) got 0 point and were divided into the low health knowledge level group. For the purpose of maximizing statistical power, we divided the others by median (i.e. 3.5). Of the other 520 participants, 243 (40.7%) were divided into moderate (1–3 points) health knowledge group, and 277 (46.4%) were divided into high (4–15 points) health knowledge group.

#### Controlling and outcome variables

Using NHI claims records and ICD-9-CM codes, we defined diabetes, hypertension, hyperlipidemia, coronary artery disease, atrial fibrillation, and congestive heart failure as at least three relevant ambulatory claims or one inpatient claim. The study cohort was defined as people without stroke at the very beginning of follow-up period. Namely, people who had received a stroke diagnosis before 2005 were excluded from current analyses. The outcome variable was stroke (ICD-9-CM codes 430–438), and the considered potential confounders were hypertension (ICD-9-CM codes 401–405), hyperlipidemia (ICD-9-CM codes 272), atrial fibrillation (ICD-9-CM codes 427.3), coronary artery disease (ICD-9-CM codes 410–414), and congestive heart failure (ICD-9-CM codes 428). Body mass index (kg/m^2^), another potential confounder, was retrieved from individual NHIS records at baseline and was categorized as underweight (<18.5), normal weight (18.5–23.9), overweight (24–26.9), and obesity (≥27) according to the definition of the Health Promotion Administration, Ministry of Health and Welfare of Taiwan [14].

### Statistical methods

Kaplan–Meier curves were firstly used to describe and examine the occurrences of stroke over time by three groups of health knowledge levels with a log-rank test. Univariate Cox proportional hazards models were then used to characterize study cohort for each of the independent variables. At last, multivariate Cox regression was applied to estimate the effect of health knowledge level to stroke with potential cofounders controlled. The test for linear trend was also performed to examine the exposure-response gradient between health knowledge levels and stroke incidence. All analyses were conducted using SAS 9.4 statistical software package (SAS Institute Inc., Cary, NC, USA).

## Results

A total of 12,157 adults aged 18 to 65 years participated in the 2005 NHIS, among whom 662 had diabetes. After excluding those with a history of stroke (*n* = 60) and those who did not respond to the health knowledge questionnaire (*n* = 5), data from 597 people with diabetes were involved in the analysis. The overall mean (SD) age was 51.28 (9.7) years old, and 51.59% of the subjects were male. Sixty-five individuals had incident stroke during 5094.8 person-years of follow-up.

**Fig. 1:**
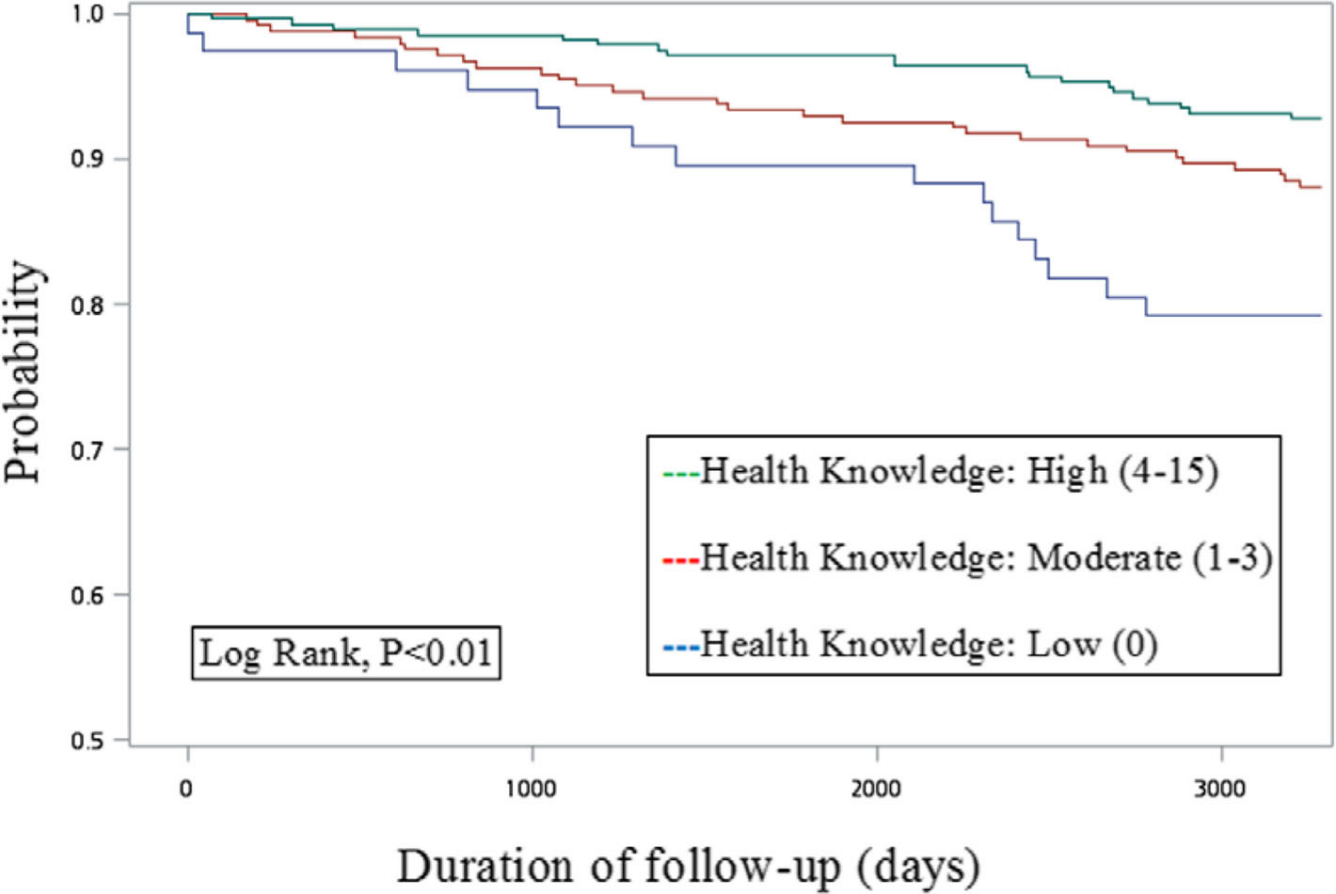
Kaplan–Meier survival curve estimates for incident stroke in people with diabetes

Kaplan–Meier curves comparing the three groups of low/moderate/high knowledge levels revealed a statistical significance (*p*-value of log-rank test <0.01; Fig. 1). During the study follow-up period (2005–2013), new-onset stroke was identified in 16 (20.78%) subjects with low health knowledge, 29 (11.93%) with moderate health knowledge, and 20 (7.22%) with high health knowledge (Table 1). As compared with individuals with low health knowledge, the relative risk of incident stroke was lower among those with moderate (HR = 0.54; 95% CI 0.30–1.00; *p*-value = 0.05) and high health knowledge (HR = 0.32; 95% CI 0.17–0.61; *p*-value < 0.01) in univariate analysis. Other factors associated with incident stroke were older age, being widowed/divorced/separated, moderate education, current smoking, atrial fibrillation, and congestive heart failure. The exposure-response relationship of health knowledge and incident diabetes was evaluated by using the trend test. The risk of incident stroke decreased as health knowledge increased (HR = 0.57; 95% CI, 0.41–0.79; *p*-value for trend < 0.01).

**Table 1:** Characteristics and results of univariate Cox regression analysis of a random community sample of people with diabetes in Taiwan (*N* = 597; 69 stroke cases)

Characteristics	Mean ± SD or number (% in column)	No. of strokes (% in row)	Hazard Ratio	95% CI	*P*-value
Health knowledge^a^					
Low (0)	77(12.90%)	16(20.78%)	1		
Moderate (1–3)	243(40.7%)	29(11.93%)	0.54	0.30–1.00	0.05
High (4–15)	277(46.4%)	20(7.22%)	0.32	0.17–0.61	<0.01
Mean (SD) age, years,	51.28(9.17)	54.63(7.64)	1.05	1.02–1.09	<0.01
Gender					
Female	289(48.41%)	31(10.73%)	1		
Male	308(51.59%)	34(11.04%)	1.02	0.63–1.66	0.93
BMI					
Underweight (<18.5)	12(2.01%)	1(8.33%)	0.67	0.09–4.99	0.70
Normal weight (18.5–24)	193(32.33%))	23(11.92%)	1		
Overweight (24–27)	182(30.49%)	20(10.99%)	0.92	0.50–1.67	0.78
Obese (>27)	205(34.34%)	20(9.76%)	0.81	0.44–1.47	0.48
Marital status					
Married/cohabiting	480(80.40%)	44(9.17%)	1		
Single	45(7.54%)	6(13.33%)	1.46	0.62–3.43	0.38
Widowed/divorced/separated	71(11.89%)	15(21.13%)	2.42	1.34–4.34	<0.01
Education					
Low (elementary or below)	274(45.90%)	39(14.23%)	1		
Moderate (junior/senior high)	231(38.69%)	18(7.79%)	0.53	0.31–0.93	0.03
High (college or above)	92(15.41%)	8(8.70%)	0.59	0.28–1.26	0.17
Household income in tertiles					
Low (<US$968/month)	181(30.32%)	22(12.15%)	1		
Moderate (US$968–2258/month)	225(37.69%)	27(12%)	0.97	0.55–1.70	0.91
High (>US$2258/month)	155(25.96%)	14(9.03%)	0.72	0.37–1.40	0.33
Smoking status					
Never	410(68.68%)	37(9.02%)	1		
Current	150(25.13%)	25(16.67%)	1.92	1.16–3.20	0.01
Former	37(6.20%)	3(8.10%)	0.91	0.28–2.94	0.87
Alcohol consumption					
None	406(68.01%)	42(10.34%)	1		
Yes	191(31.99%)	23(12.04%)	1.16	0.70–1.93	0.57
Comorbidities					
Hypertension					
No	179(29.98%)	14(7.82%)	1		
Yes	418(70.02%)	51(12.2%)	1.58	0.88–2.86	0.13
Hyperlipidemia					
No	180(30.15%)	14(7.78%)	1		
Yes	417(69.85%)	51(12.23%)	1.60	0.89–2.89	0.12
Atrial fibrillation					
No	584(97.82%)	60(10.27%)			
Yes	13(2.18%)	5(38.46%)	4.97	1.99–12.39	<0.01
Coronary artery disease					
No	401(67.17%)	36(8.98%)	1		
Yes	196(32.83%)	29(14.8%)	1.70	1.04–2.78	0.03
Congestive heart failure					
No	551(92.29%)	55(9.98%)	1		
Yes	46(7.71%)	10(21.74%)	2.33	1.19–4.57	0.01

*Abbreviations*: *CI* confidence interval, *BMI* body mass index

^a^The dose–response relationship of health knowledge and incident stroke was evaluated by the trend test (HR, 0.57; 95% CI, 0.41–0.79; *P* for trend < 0.01)

A multivariate Cox regression model was used to estimate the effect of health knowledge level to the risk of stroke in people with diabetes with potential confounders considered (Table 2). Education variable was excluded from the final model because of its high correlation to health knowledge level, the major exposure of interest in current analysis. After controlling for subject demographics and comorbidities, the risk of stroke incidence was lower for those with moderate (adjusted hazard ratio, AHR = 0.63; 95% CI, 0.33–1.19; *p*-value = 0.15) and high health knowledge (AHR = 0.43; 95% CI, 0.22–0.86; *p*-value = 0.02), comparing to the group with low health knowledge. A statistically significant exposure-response gradient of health knowledge to the incidence of stroke by a test for linear trend remained (*p*-value = 0.02; Table 2).

**Table 2:** Results of multivariate Cox proportional hazards analysis of incident stroke risk among people with diabetes

Characteristics	HR	95% CI	*P*-value
Health knowledge^a^			
Low (0)	1		
Moderate (1–3)	0.63	0.33–1.19	0.15
High (4–15)	0.43	0.22–0.86	0.02
Age	1.04	1.00–1.07	0.04
Sex	0.81	0.42–1.58	0.54
Smoking status			
Never	1		
Current	2.24	1.13–4.44	0.02
Former	0.92	0.25–3.34	0.90
Comorbidities			
Hypertension			
No	1		
Yes	0.96	0.50–1.85	0.91
Hyperlipidemia			
No	1		
Yes	1.88	1.00–3.55	0.05
Atrial fibrillation			
No	1		
Yes	3.61	1.28–10.20	0.02
Coronary artery disease			
No	1		
Yes	1.16	0.67–2.01	0.60
Congestive heart failure			
No	1		
Yes	1.61	0.75–3.45	0.22

*Abbreviations*: *HR* hazard ratio, *CI* confidence interval

^a^The dose–response relationship of health knowledge and incident stroke was evaluated by the trend test (AHR, 0.66; 95% CI, 0.47–0.93; *P* for trend = 0.02)

## Discussion

Our findings show that people with type 2 diabetes mellitus with a higher level of health knowledge possessed a lower risk of incident stroke during the 9-year follow-up period. People with awareness of health knowledge may be more aggressive in behavior change by means of better drug compliance, regular exercise, adequate nutrition, limiting salty and high-fat foods, and blood pressure and glucose control. The P4P program is a payment-based model with financial incentives to healthcare providers to meet the pre-established medical services delivery clinical goals, [15] which consisted of full follow-up visits including intensive self-care education program as well as regular diabetes-specific physical examinations, including ophthalmic and laboratory examinations (e.g. hemoglobin A1c, renal function, proteinuria, and lipid profiles) [16]. Evidence about the improvement of glycemic control and lipid profiles by the performance of P4P schemes was reported [17]. P4P programs for diabetes in Taiwan were associated with significant increases in regular follow-up visits and use of evidence-based services and significantly lower hospitalization costs. Evidence of potential long-term benefits of P4P programs in reducing risks of macrovascular complications in diabetes was reported [12, 18]. However, enrollment in a P4P program does not guarantee awareness of health knowledge. Few investigated the association of awareness of health knowledge with development of macrovascular complications in people with diabetes in community settings.

Diabetic mortality was associated with socioeconomic status, including education level and household income. A study in Europe showed that education and diabetes mortality had an inverse association in both genders [19]. People with a higher education level had lower diabetes mortality rates [19]. Saydah et al. investigated diabetes-related mortality for different socioeconomic status groups with nationally representative samples in the USA. The result showed that having less than a high school education or household income below the defined poverty line was associated with a higher mortality [20]. Further analysis on the relative and absolute disparities in education revealed that the risk of all-cause mortality was higher in diabetic patients who had the lowest educational level [18].

Absolute education-related inequalities also lead to total mortality of cardiovascular disease in Spanish adults [21]. There are several crucial factors associated with health knowledge awareness. Hamner et al. had reported that people with health insurance and were employed full time had higher knowledge scores [22]. Potvin et al. reported that education is one of the essential indicators of socioeconomic status, which is closely related to knowledge of cardiovascular risk factors [23]. Our study also showed that education variable was highly correlated to health knowledge level. Education level was not easy to change in adult population, but awareness of health knowledge can be enhanced by community health education to prevent long-term morbidity and mortality.

Our study also showed that health knowledge of people with diabetes in Taiwan is limited. In current analysis, we found that 77 (12.9%) people with diabetes had very poor health knowledge and that 243 (40.7%) others had scores lower than 4 out of 15 points. The knowledge level of recognition, risk factors, and symptoms of stroke in US and Austrian population were reported low [24, 25]. A comprehensive systematic review of 39 related studies on knowledge and awareness of stroke showed that both the knowledge of recognizing and preventing stroke were poor [26]. People with older age, minority ethnical groups, and those with a lower education level had a lower level of stroke knowledge [26]. Community health education workshops may improve health knowledge and behavior. Implementing community-based education in a customized curricula can improve the health knowledge and behaviors of community residents and reduce cardiovascular disease risks [27]. Community-based lifestyle management program for people with DM was reported to lead to short-term beneficial changes in physical activity, nutrition, and laboratory parameters [28, 29]. There are many diabetes education program for delivering health knowledge to people with diabetes, including verbal, written, and other visual information and repeated educational encounters [30]. Physicians, nutritionists, and educators are the primary sources of health information. In addition, the mass media, especially television, was reported to be a source of health knowledge for most respondents [31, 32]. Provision of appropriate community health care services and health education are essential to improve population health because of rapidly increasing chronic diseases related to aging, obesity, and lifestyle behaviors. Therefore, local community health authorities should increase the manpower of technical staff and restructure the community health centers to strengthen health education among people in community.

The present study has limitations that warrant further notices. We analyzed self-reported information, including weight, height, household income, smoking status and alcohol consumption, which might have introduced information bias. Although the percentage of refusing to provide an informed consent to access to their national health insurance data was low, the concern about selective missing for the detection of outcomes still exists [33]. In addition, deaths were not recorded in the NHI database. However, because the mean age of the study subjects was 51.28 years and the mean duration of follow-up was 9 years, this bias would be small due to the low mortality in this population during the follow-up period. Because of limited availability of certain data, the assessment of health knowledge was only performed in the very beginning of follow up, which did not reflect the real situation of potential health knowledge level change over time. The duration between diabetes diagnosis and interview for health knowledge measurement in 2005 was not available for analysis. Finally, laboratory data such as blood glucose levels, hemoglobin A1c levels, lipid profiles, and blood pressure levels were not available for analysis.

A main strength of our study is its retrospective cohort design, which abstains the concern of control selection in case–control studies and ambiguous temporality in cross-sectional studies. We analyzed data from a nationally representative sample of the general population in Taiwan, which provided greater generalizability and external validity. The NHIS was designed and administered by a well-trained national survey team with interview quality control. The study cohort was followed up as long as 9 years, providing sufficient person-years to obtain quite many stroke subjects and a certain level of statistical power. Finally, we adjusted for several important confounding variables, including age, gender, body mass index, smoking habits, drinking, and comorbidities, which were not achievable for many other studies related research fields.

Our study revealed that people with diabetes mellitus can reduce risks of stroke incidence through obtaining more health knowledge. Stroke stands for the third leading cause of death in Taiwan, and it is also the most common cause of complex disability [34]. The burden of stroke can be decreased by the development and delivery of stroke prevention projects, which could be achieved by applying effective primary and secondary prevention approaches at both individual and population levels. Conducting educational campaigns to raise health knowledge awareness among the general population, and delivering targeted information to high-risk groups and their families may help to reduce stroke risks for people with type 2 diabetes mellitus.

## Conclusions

The present results highlight the importance of developing and delivering education campaigns on stroke prevention to people with diabetes in community setting. Our findings pinpointed the critical role of delivering health knowledge to reduce the occurrence of stoke among specific vulnerable groups and minimize the burden of it to human society.

## Abbreviations

ICD-9-CM: International Classification of Diseases, Ninth Revision, Clinical Modification
NHI: National Health Insurance
NHIS: National Health Interview Survey
P4P: Pay-for-performance
